# Supplementary material for: Research and application of OPC UA-based digital twin smoke alarm calibration workshop information model
Source: PLoS One. 2024 Jul 25;19(7):e0307801. doi: 10.1371/journal.pone.0307801 (PMC11271951; doi:10.1371/journal.pone.0307801)
Supplement: Attachment — Submitted filename: PONE-D-24-16301 (the marked manuscript).pdf [file pone.0307801.s001.pdf]

PLOS ONE

Research and application of OPC UA-based digital twin smoke alarm calibration workshop information model

--Manuscript Draft--

**Manuscript Number:**	PONE-D-24-16301
**Article Type:**	Research Article
**Full Title:**	Research and application of OPC UA-based digital twin smoke alarm calibration workshop information model
**Short Title:**	OPC UA-based digital twin smoke alarm calibration workshop information model
**Corresponding Author:**	min wu<br>Ningbo Polytechnic<br>Ningbo, CHINA
**Keywords:**	OPC UA; Smoke alarm; Calibration workshop; Information model; Smoke box
**Abstract:**	Aiming at the problems of data integration and information sharing in the calibration workshop of smoke alarm, combining OPC UA and digital twin technology, the OPC UA based digital twin smoke alarm calibration workshop information network framework is proposed. Using OPC UA information modeling technology, a digital workshop information model for smoke alarm calibration was constructed, and the static attribute set, process attribute set, and functional component set of the workshop were defined in detail. Taking the smoke box as an example, the information model construction was instantiated, and data collection and transmission were carried out with the digital twin model of the smoke box, achieving the implementation mapping between the twin model and physical entities. The results show that based on the proposed OPC UA information model, the interconnection and intercommunication of data at all levels of the digital twin smoke alarm calibration workshop can be achieved, and the workshop can be visualized, digitized, and flexible.
**Order of Authors:**	min wu
	Wen feng Ying
**Additional Information:**	
**Question**	**Response**
**Financial Disclosure**<br><br>Enter a financial disclosure statement that describes the sources of funding for the work included in this submission. Review the submission guidelines for detailed requirements. View published research articles from *PLOS ONE* for specific examples.<br><br>This statement is required for submission and **will appear in the published article** if the submission is accepted. Please make sure it is accurate.	Yes

Powered by Editorial Manager® and ProduXion Manager® from Aries Systems Corporation

**Funded studies**<br>Enter a statement with the following details:<br>• Initials of the authors who received each award<br>• Grant numbers awarded to each author<br>• The full name of each funder<br>• URL of each funder website<br>• Did the sponsors or funders play any role in the study design, data collection and analysis, decision to publish, or preparation of the manuscript?<br><br>Did you receive funding for this work?	
Please add funding details.<br>as follow-up to "**Financial Disclosure**<br><br>Enter a financial disclosure statement that describes the sources of funding for the work included in this submission. Review the submission guidelines for detailed requirements. View published research articles from *PLOS ONE* for specific examples.<br><br>This statement is required for submission and **will appear in the published article** if the submission is accepted. Please make sure it is accurate.<br><br>**Funded studies**<br>Enter a statement with the following details:<br>• Initials of the authors who received each award<br>• Grant numbers awarded to each author<br>• The full name of each funder<br>• URL of each funder website<br>• Did the sponsors or funders play any role in the study design, data collection and analysis, decision to publish, or preparation of the manuscript?<br><br>Did you receive funding for this work?"	This work was supported by 2022 Visiting Engineer of Colleges and Universities in Zhejiang Province(Project No. FG2022037).
Please select the country of your main research funder (please select carefully as in some cases this is used in fee calculation).	CHINA - CN

as follow-up to "**Financial Disclosure**<br><br>Enter a financial disclosure statement that describes the sources of funding for the work included in this submission. Review the submission guidelines for detailed requirements. View published research articles from *PLOS ONE* for specific examples.<br><br>This statement is required for submission and **will appear in the published article** if the submission is accepted. Please make sure it is accurate.<br><br>**Funded studies**<br>Enter a statement with the following details:<br>• Initials of the authors who received each award<br>• Grant numbers awarded to each author<br>• The full name of each funder<br>• URL of each funder website<br>• Did the sponsors or funders play any role in the study design, data collection and analysis, decision to publish, or preparation of the manuscript?<br><br>Did you receive funding for this work?"	
**Competing Interests**<br><br>Use the instructions below to enter a competing interest statement for this submission. On behalf of all authors, disclose any competing interests that could be perceived to bias this work—acknowledging all financial support and any other relevant financial or non-financial competing interests.<br><br>This statement is required for submission and **will appear in the published article** if the submission is accepted. Please make sure it is accurate and that any funding sources listed in your Funding Information later in the submission form are also declared in your Financial Disclosure statement.	he authors have declared that no competing interests exist.

View published research articles from *PLOS ONE* for specific examples.<br><br>**NO authors have competing interests**<br><br>Enter: *The authors have declared that no competing interests exist.*<br><br>**Authors with competing interests**<br><br>Enter competing interest details beginning with this statement:<br><br>*I have read the journal's policy and the authors of this manuscript have the following competing interests: [insert competing interests here]*<br><br>* typeset	
**Ethics Statement**<br><br>Enter an ethics statement for this submission. This statement is required if the study involved:<br><br>• Human participants<br>• Human specimens or tissue<br>• Vertebrate animals or cephalopods<br>• Vertebrate embryos or tissues<br>• Field research<br><br>Write "N/A" if the submission does not require an ethics statement.<br><br>General guidance is provided below. Consult the submission guidelines for detailed instructions. **Make sure that all information entered here is included in the Methods section of the manuscript.**	N/A

Powered by Editorial Manager® and ProduXion Manager® from Aries Systems Corporation

**Format for specific study types**<br><br>**Human Subject Research (involving human participants and/or tissue)**<br>• Give the name of the institutional review board or ethics committee that approved the study<br>• Include the approval number and/or a statement indicating approval of this research<br>• Indicate the form of consent obtained (written/oral) or the reason that consent was not obtained (e.g. the data were analyzed anonymously)<br><br>**Animal Research (involving vertebrate animals, embryos or tissues)**<br>• Provide the name of the Institutional Animal Care and Use Committee (IACUC) or other relevant ethics board that reviewed the study protocol, and indicate whether they approved this research or granted a formal waiver of ethical approval<br>• Include an approval number if one was obtained<br>• If the study involved *non-human primates*, add *additional details* about animal welfare and steps taken to ameliorate suffering<br>• If anesthesia, euthanasia, or any kind of animal sacrifice is part of the study, include briefly which substances and/or methods were applied<br><br>**Field Research**<br><br>Include the following details if this study involves the collection of plant, animal, or other materials from a natural setting:<br>• Field permit number<br>• Name of the institution or relevant body that granted permission	
**Data Availability**<br><br>Authors are required to make all data underlying the findings described fully available, without restriction, and from the time of publication. PLOS allows rare exceptions to address legal and ethical concerns. See the PLOS Data Policy and FAQ for detailed information.	Yes - all data are fully available without restriction

A Data Availability Statement describing where the data can be found is required at submission. Your answers to this question constitute the Data Availability Statement and **will be published in the article**, if accepted.<br><br>**Important:** Stating 'data available on request from the author' is not sufficient. If your data are only available upon request, select 'No' for the first question and explain your exceptional situation in the text box.<br><br>Do the authors confirm that all data underlying the findings described in their manuscript are fully available without restriction?	
**Describe where the data may be found in full sentences. If you are copying our sample text, replace any instances of XXX with the appropriate details.**<br><br>• If the data are **held or will be held in a public repository**, include URLs, accession numbers or DOIs. If this information will only be available after acceptance, indicate this by ticking the box below. For example: *All XXX files are available from the XXX database (accession number(s) XXX, XXX.)*.<br>• If the data are all contained **within the manuscript and/or Supporting Information files**, enter the following: *All relevant data are within the manuscript and its Supporting Information files.*<br>• If neither of these applies but you are able to provide **details of access elsewhere**, with or without limitations, please do so. For example:<br><br>*Data cannot be shared publicly because of [XXX]. Data are available from the XXX Institutional Data Access / Ethics Committee (contact via XXX) for researchers who meet the criteria for access to confidential data.*<br><br>*The data underlying the results presented in the study are available from (include the name of the third party*	All relevant data are within the manuscript and its Supporting Information files.

and contact information or URL).<br>• This text is appropriate if the data are owned by a third party and authors do not have permission to share the data.<br><br>* typeset	
Additional data availability information:	

Manuscript Click here to access/download;Manuscript;manuscript(p).docx

Research and application of OPC UA-based digital twin smoke alarm calibration workshop information model

Wu Min[1]*, *Ying Wenfeng*[2]

1.School of mechanical and electrical engineering, Ningbo Polytechnic, Ningbo , Zhejiang,China

2. Ningbo weizmat Electronics Co., Ltd., Ningbo ,Zhejiang,China

*Corresponding author:53760012@qq.com

Abstract

Aiming at the problems of data integration and information sharing in the calibration workshop of smoke alarm, combining OPC UA and digital twin technology, the OPC UA based digital twin smoke alarm calibration workshop information network framework is proposed. Using OPC UA information modeling technology, a digital workshop information model for smoke alarm calibration was constructed, and the static attribute set, process attribute set, and functional component set of the workshop were defined in detail. Taking the smoke box as an example, the information model construction was instantiated, and data collection and transmission were carried out with the digital twin model of the smoke box, achieving the implementation mapping between the twin model and physical entities. The results show that based on the proposed OPC UA information model, the interconnection and intercommunication of data at all levels of the digital twin smoke alarm calibration workshop can be achieved, and the workshop can be visualized, digitized, and flexible.

Keywords: OPC UA; Smoke alarm; Calibration workshop; Information model; Smoke box

1Introduction

With the successive propositions of strategies such as Industry 4.0 and Made in China 2025, intelligent manufacturing has become the core of development for countries at the advent of the fourth industrial revolution. As the best means of integrating interactive physical world and virtual world, digital twin is a major driving force to promote the further transformation of manufacturing workshop towards intelligence, personalization and service[1]. With the increasing demand for smoke alarms, their quantity and variety are also increasing. The calibration workshop of smoke alarms will generate massive data during production and operation. How to use these data to establish a multi-domain integrated data model and achieve an efficient application model of real-time interaction between data model and physical entity is a problem to be solved in the production process of smoke alarms[2]. Digital twin technology provides a possibility to solve this problem[3].

The core of digital twinning is modeling and data interaction[4]. Designing a reasonable information-physical fusion system for digital twinning workshop is the foundation for building virtual-real interaction in digital workshop[5], and it is also the key to solving the problem of information island in digital workshop. OPC UA (OPC Unified Architecture) protocol is one of the current common data transmission standards in the industrial field, which realizes the semantic unification of data between various layers in intelligent manufacturing workshop[6]. Regarding the application of OPC UA technology, Song[7] et al. implemented data integration and unified management of intelligent workshop equipment information based on OPC UA technology. Li[8] et

al. proposed a textile intelligent dyeing and finishing workshop information model scheme based on OPC UA technology, which realized data communication between equipment end and manufacturing execution system. Wang[9] et al. proposed a worksheet definition format (WDF) file model based on OPC UA, which can realize open sharing of information interaction between various levels in digital manufacturing workshop of CNC machine tools and solve the problem of information heterogeneity. Zhou[10] et al. designed a network acquisition server based on OPC UA standard architecture to solve the networking problem of various CNC equipment. Chen[11] et al. implemented three-dimensional real-time monitoring of digital twinning workshop operation based on multi-source heterogeneous data acquisition and transmission mode in workshop based on OPC UA technology.

In summary, this article studies the information model of the digital twin smoke alarm calibration workshop based on OPC UA, constructs an OPC UA information interaction network architecture for the hoist system based on four types of multi-source and multi-dimensional heterogeneous data including people, machines, objects, and environment, analyzes the functional models and information flows that meet the requirements of the smoke alarm calibration workshop, and proposes an information modeling method applicable to the smoke alarm calibration workshop. The feasibility of the information model of the smoke alarm calibration workshop is verified through the built digital twin system platform.

2 OPC UA information modeling technology

The OPC Unified Architecture provides a standardized, synchronous or asynchronous, and distributed communication mechanism. Under this mechanism, it allows access to different types of data horizontally or vertically. OPC UA components can be combined in different forms, on different platforms, and by different vendors. Their scale can range from embedded OPC UA components in devices or controllers, to machines or complete sets of equipment using network management to provide OPC UA functionality, all the way to OPC UA server clusters. OPC UA is generally based on a distributed client-server model.

2.1 OPC UA Server

According to the functional requirements of the digital twin smoke alarm calibration workshop, an aggregated soft OPC UA server architecture is adopted, as shown in Fig1. The aggregated OPC UA server unifies the OPC UA servers for each asset, incorporates the information models of all servers in the lower layer, and integrates them at the system aggregation level. This architecture type can be connected to enterprise MES or ERP, or used to separate manufacturing systems from later data analysis or earlier planning. The OPC UA client can be either a SCADA, MES, ERP, or central control system maintained by the IT department, a visualization terminal running near the machine, or even an external application.

2.2 OPC UA address space and information model

The object model of OPC UA allows the integration of data, alarms, events, and historical data into an OPC UA server address space. The objects and related information provided by the OPC UA server to the client are called the address space. The address space of the OPC UA server is a fully interconnected information model that is presented in a graph-like topology[12]. This information model includes nodes, the characteristics of the nodes themselves, and the interconnections between nodes. OPC UA defines

eight node classes, each with a defined set of properties. Any node in OPC UA can be connected to each other. The OPC UA server provides the client with object type definitions that are accessed from the address space, and an information model to describe standardized nodes in the server address space.

Fig1 .Aggregated Soft OPC UA Architecture

2.3 OPC UA application architecture

Fig2 shows the application architecture of OPC UA client and server. OPC UA uses client/server mode to implement information exchange. The client application uses OPC UA client API to send and receive service requests and responses from OPC UA server. The server application uses OPC UA server API to send and receive messages from OPC UA client. Clients can access nodes in the server using OPC UA services [13].

Fig2. OPC UA client and server application architecture

2.4 Digital workshop information model

This article establishes a digital workshop information model for smoke alarms based on the operating mode and organizational structure of digital production workshops. The hierarchical architecture of the workshop information model is shown in Fig3. The workshop information model corresponds to the entire workshop, the functional component set corresponds to each functional module of the workshop, the resource component set represents various resources involved in each module, and the attribute set represents the collection of attributes. Attributes are the basic units described by the information model, with a fixed structure, used to describe the association between equipment, components, attribute sets, and attributes[14].

Fig3. Workshop information model hierarchy

3. Construction of digital twin smoke alarm calibration workshop information model

3.1 Digital twin smoke alarm calibration workshop information network architecture

The data integration and fusion of multi-source heterogeneous data in the digital twin workshop are the connection channels to realize the control and management of the production workshop and to open up the interactive mapping between the virtual and real workshop. Based on the operation mode of the digital twin workshop and the requirements of virtual and real integration, an OPC UA-based digital twin smoke alarm calibration workshop information network architecture is proposed. According to the functional requirements of the workshop, the system architecture is divided into three layers: application layer, processing layer, and perception layer, as shown in Fig4.

Fig4. Information network architecture of digital twin smoke alarm calibration workshop

The perception layer consists of PLC, smoke box, various sensors, industrial robots, AGV, RFID and other devices. These devices are used to collect workshop data. Most of the on-site perception devices have integrated OPC UV servers internally, which can be connected to the OPC UV server through internal communication. For devices without built-in OPC UV servers, communication with the OPC UV server can be achieved through gateways, middleware, routers, etc.

The processing layer establishes a standardized information model for multi-source heterogeneous data generated by different devices in the sensing layer through the OPC UV communication protocol. Based on the characteristics of OPC UA modeling and transmission, the standardized information model is combined with the address space module and the collected normalized data to construct the address space of the OPC UA server, and the information model is instantiated. Then, through the OPC UA server, the address space data mapping between the physical model and the twin model is completed, and the OPC UA client is directly integrated into the digital twin service platform, providing a unified communication architecture for digital twin service and data collection.

The application layer is the application program that obtains the address space of the aggregated soft OPC UA server. The OPC UA client application communicates with the aggregated soft OPC UA server in the processing layer through the OPC UA protocol, including digital twin software, workshop twin database, information system, and workshop service system. The digital twin software provides a simulation environment and uses OPC UA communication to achieve virtual-real mapping. The information system obtains data information and provides data analysis and production plan control and adjustment.

3.2 Construction of the information model for the calibration workshop of the digital twin smoke alarm

Refer to GB/T 38869-2020 OPC UA based Digital Workshop Interconnection Network Architecture and GB/T 37928-2019 Digital Workshop Machine Tool Manufacturing Information Model, sort out the integrated information of the smoke alarm calibration workshop, and use OPC UA modeling method to obtain the smoke alarm calibration workshop information model, as shown in Fig5.

Fig5. Information model of smoke alarm calibration workshop

The information model for the smoke alarm calibration workshop mainly includes static attribute sets, process attribute sets, and functional component sets. The functional component sets include four sub-models: production operation management information model, maintenance operation management information model, quality operation management information model, and logistics operation management information model[15]. Each sub-model includes its own static attribute sets, process attribute sets, and resource component sets.

The smoke alarm calibration workshop static attribute set defines the basic static information of the workshop operation, including the basic information of the workshop (such as the name of the workshop, the location of the workshop, the area of the workshop, the person in charge of the workshop, etc.), production organization (such as the departments and work groups in the workshop), work calendar (such as the working days and rest days of the workshop), and smoke alarm calibration work orders (order information obtained by the MES system from the ERP system). The process attribute set defines the dynamic data of the workshop process, which is generally summarized statistical data and order tracking information of various aspects of the workshop, and is used to provide feedback to the ERP system, such as order tracking, calibration statistics, quality statistics, inventory statistics, maintenance statistics, etc. [16].

The static attribute set of each sub-model in the functional component set defines the set of static attribute data related to the implementation of each functional module in the smoke alarm calibration workshop. The static attribute data refers to data that does not change or changes slowly over a certain period of time. The process attribute set defines the data collected during the calibration process that may

change as the calibration process progresses. The resource component set defines the information model of the equipment, tooling, and personnel that make up the smoke alarm workshop.

The production operation management information model process attribute set defines the information objects and their data attributes generated during the smoke alarm calibration process, including calibration plan tracking, calibration work order dispatch, smoke box operation information, and actual job information. The production resource component set of the production operation management information model defines the information models related to equipment, tooling, personnel, etc. for smoke alarm calibration, including smoke boxes, robots, AGV cars, calibration personnel, etc.

The static attribute set of the smoke alarm calibration workshop maintenance operation management information model defines the static information set involved in daily management and maintenance of equipment, including equipment account, equipment maintenance specifications, equipment point inspection specifications, etc. The process attribute set of the maintenance operation management information model defines the information objects and their attribute sets generated during the maintenance process of equipment, including equipment repair, equipment maintenance, equipment point inspection, equipment failure, equipment alarm, equipment performance, etc. The maintenance resource component set includes maintenance tools, maintenance equipment, and maintenance personnel information.

The static attribute set of the smoke alarm calibration workshop quality operation management information model defines the static information set involved in the quality inspection of the alarm, including various alarm sensitivity standards, quality inspection plans, and defect definitions. The process attribute set of the quality operation management information model defines the information objects and their attribute sets generated by the execution of the quality inspection process and its results, including quality inspection dispatch orders, quality inspection execution, quality inspection certificates, non-conforming product reports, rework and repair orders, quality inspection plan tracking, and scrap product processing. The resource component set of the quality operation management information model includes information such as quality inspection personnel, quality inspection equipment, and quality inspection tools.

The static attribute set of the smoke alarm calibration workshop logistics operation management information model defines the static information set related to logistics management, outbound and inbound warehousing, and distribution and shipping during the operation process of the workshop, including material basic information, material procurement plan, material production plan, etc. The process attribute set of the logistics operation management information model defines the information model objects and their attribute sets generated by dynamic behaviors such as material allocation and inbound and outbound warehousing involved in the calibration process of the alarm, including material outbound, material inbound, material transfer, material inventory, material return, material distribution, etc. The resource component set of the logistics operation management information model includes warehouse information model, logistics equipment information model, logistics tool information model, etc.

4 Application of information model in calibration workshop

4.1 Introduction to the calibration workshop of Wizmart

With the increasing demand for digitalization and intelligence in workshop production, Ningbo

Weizmat Electronics Co., Ltd. has created a digital sample workshop for smoke alarm calibration. The workshop consists of 10 sets of automated smoke boxes, 10 automatic labeling machines, 10 automatic coding machines, 10 sets of horizontal SCARA robot automated production lines, and some auxiliary equipment. The production line integrates high-end intelligent equipment such as automatic warehousing, loading and unloading, detection, labeling, and coding, and integrates with software systems such as product lifecycle management, enterprise resource planning systems, and production line control to achieve automatic warehousing, automatic loading and unloading, automatic detection, automatic calibration, automatic logistics operation, barcode information tracking, and other automated production for smoke alarms. The entire production line has achieved visualization, intelligence, and flexibility, completely changing the backward production mode of manual detection[17].

The process flow for calibrating the smoke alarm is: using an AGV car for the product - workshop material warehouse - feeding - conveying (distributing) - horizontal SCARA robot picking up material - recording coordinates with a camera - placing the product in the fixture position - entering the smoke box - automatically setting the PPM value of the smoke box - communicating with the product - telling the product the current PPM value and calibrating it - exiting the warehouse - horizontal SCARA robot picking up material - reading the QR code - recording - placing the finished product in the channel - conveying - workshop material warehouse.

4.2 Information Model Instantiation

4.2.1 Modeling of the smoke box information model

Due to the complexity of the information model for the calibration workshop of smoke alarms, the smoke box is the most important device for calibrating the workshop. This article focuses on the instantiation of the information model with the smoke box as the object. The standard smoke box consists of several components, physical attributes, and various operations. Each component contains other subcomponents and physical attributes, so it is necessary to define relevant information model elements to abstract and describe the smoke box. Fig6 shows the mapping between the smoke box and information model elements.

Fig6. Mapping between the smoke box and the information model element

The information model of the smoke box is shown in Fig7. The standard smoke box information model consists of a static attribute set, a process attribute set, and a component assembly set. The component assembly set mainly consists of four subcomponents: a transmission device, a smoke generating device, an airflow regulating device, and a measuring device. The subcomponent information model includes static and process attributes. The static attribute set of the smoke box information model mainly includes the basic information of the smoke box (box name, model, number, manufacturer, specifications, etc.), technical parameters (airflow speed, smoke generation rate, input voltage, measurement error, humidity, temperature, etc.), functional indicators (response threshold calibration, response threshold experiment, consistency experiment, orientation experiment, repeatability experiment, etc.), and process attributes include operation information (calibration alarm name, calibration alarm model, calibration alarm order number, operation technical parameters, etc.), alarm information (unqualified alarm name, unqualified alarm model, unqualified alarm order number, environmental parameter overrun alarm information, equipment failure alarm, etc.), and shift production information (shift number, personnel on duty, production time, downtime). The static attribute set of the transmission device information model includes the number, state, motor name, cylinder name, etc., and

the process attributes include frequency conversion current, motor speed, cylinder speed, position sensor status, product position, etc. The static attribute set of the smoke generating device information model includes the number, state, experimental smoke variety, and the process attributes include smoke particle size, smoke generation rate, smoke production volume, experimental smoke stability, smoke generation control mode, etc. The static attribute set of the airflow regulating device information model includes the number, state, fan name, fan quantity, fan name, fan control mode, electric hanging basket name, electric hanging basket control. The process attributes include fan speed, fan speed, servo motor speed, hanging basket azimuth angle, airflow speed, temperature and humidity, shielding rate. The static attribute set of the measuring device information model includes the number, state, measurement range, measurement error, resolution ratio, light source, receiver, etc., and the process attributes include optical measurement length, beam wavelength, response time, product response, light sensor data.

Fig7. Information model of smoke box

4.2.2 Address Space Mapping

The description file of the smoke box is described using standard XML syntax. The information model loader parses the input XML file, maps different information model elements to the OPC UA model, and automatically generates address spaces. The mapping between smoke box information and OPC UA includes structural data mapping and pure data mapping. Structural data is for organizational structure relationships, not actual data. This type of structural information can be represented by the OPC UA “FloderType” object type. Pure data refers to the information represented by attributes. The mapping of the OPC UA model is determined based on different data types and included attribute elements. Generally, the state quantity of two values is represented by “TwoDiscrteStateType”, multiple state values are represented by”MultiStateType”, analog quantities are represented by “AnalogItemType”, and others are represented by “DataItemType”. The mapping between smoke box information models is shown in Table 1 (due to the large amount of content, only a portion is selected).

Table 1. Mapping of the information model of the smoke chamber

Number	Information model element point	OPC UA meta-model types	Citation relationship	Remarks
1	Smoke box	FloderType	Reference under the root node	
2	Static attributes	FloderType	There is an organizational reference under the smoke box node	
3	Basic Information	DataItemType	There are attribute references under the static attribute node	
4	Technical Parameter	DataItemType	There are attribute references under the static attribute node	
5	Functional Index	DataItemType	There are attribute references under the static attribute node	
6	Procedure Attribute Set	FloderType	There is an organizational reference under the smoke	

			box node	
7	Running State	MultiStateType	There are attribute references under the process attribute node	This can use multiple states, such as running, stopping, and failure.
8	Alarm Information	TwoDiscrteStateType	There are attribute references under the process attribute node	
9	Shift Production Information	AnalogItemType	There are attribute references under the process attribute node	Since this value is writable, it can be processed accordingly according to the analog output
10	Component Assembly Set	FloderType	There is an organized reference under the smoke box node	
11	Transmission Device Information Model	FloderType	There are organizational references under the component node	
12	Procedure Attribute Set	FloderType	Organized references under the transmission equipment information model node	
13	Variable Frequency Current	AnalogItemType	There are organized references under the process attributes of the transmission equipment information model node	Because this value is read-only, it can be processed accordingly according to the analog output
14	Motor Speed	AnalogItemType	Organized references are available under the process attributes of the transmission equipment information model node	Because this value is read-only, it can be processed accordingly according to the analog output
15	Cylinder Speed	AnalogItemType	Organized references under the transmission equipment information model process attribute node	
16	Position Sensor Status	TwoDiscrteStateType	There are organized references under the process attributes of the transmission equipment information model node	
17	....			

4.2.3 Address space management

The address space mainly implements corresponding read, write, and subscribe operations based on the established mapping table, as shown in Fig8. When the memory data point changes, it is updated to the OPC UA address space. When the OPC UA client reads node data, the corresponding node data is directly returned from the address space. When the OPC UA client writes data, the system provides a mechanism to ensure that the update of the memory point is associated with the actual device IO. When the OPC UA client subscribes to a node, the system can provide a mechanism to maintain the correspondence between the information of the memory point and the change of the node value in the OPC UA address space.

Fig8. Address Space Management

4.2.4 Application of digital twin smoke box

This article uses Siemens NX platform to construct a digital twin model for the smoke box, builds a virtual-reality communication platform using OPC UA communication protocol, and finally uses real-time mapping technology to realize 3D model visualization and virtual production visualization. Siemens NX is equipped with an OPC UA client, which can realize data exchange between the digital twin model's data, signals, and variables and external servers. The entire environment consists of three main components: PLC CPU, OPC server, and digital twin model (MCD). The CPU is used to process the logic of operation, obtain the machine state through the OPC server, and send operating instructions to the device through the OPC server; MCD is used to display the virtual model and perform simulation, obtain CPU instructions from OPC, and feedback the current device state through the OPC server. The data interaction between the digital twin model and physical equipment is shown in Fig9[3], and Fig10 is a comparison diagram of the digital twin model of the smoke box and the physical object.

Fig9. Data interaction between digital twin model and physical equipment

Fig10. Comparison between the digital twin model of the smoke chamber and the physical object

Through testing and verification of the digital twin system, the calibration process of the smoke box is visually monitored. The system operation is shown in Fig11. The digital twin platform provides visual monitoring, motion information, real-time data collection, and attribute information of the smoke box equipment. The production management system provides detailed information such as smoke box equipment management, equipment status, and equipment operation.

Fig11. System operation verification

5 Conclusion

As a key technology for the digital development of enterprises, digital twin technology is the key to information exchange between the physical world and the virtual world. This article applies OPC UA technology to build a virtual-real information interaction platform in the digital twin workshop. Based on the unified architecture of OPC UA, an information model for the digital workshop is proposed. Based on the operation mode of the digital twin workshop and the requirements of virtual-real fusion, an OPC UA-based digital twin smoke alarm calibration workshop data acquisition system architecture is proposed, providing an architectural foundation for multi-source heterogeneous data acquisition in the smoke alarm calibration workshop. The information model of the smoke alarm calibration workshop is constructed from three dimensions:

static attribute set, process attribute set, and functional component set, and applied to the calibration digital prototype workshop of Ningbo Weizmat Co., Ltd. Four subcomponent information models of transmission device, smoke generating device, airflow regulating device, and measuring device are constructed and analyzed in detail. After instantiating the information model, the feasibility of the model is verified using an OPC UA server, and data acquisition and transmission are performed with a digital twin platform to achieve the implementation mapping between the twin world and the physical world. This study provides strong technical support for realizing visualization, intelligence, and flexibility in smoke alarm calibration, and provides technical support and reference for realizing intelligent production workshops for smoke alarm production.

Supporting information

S1 Fig . Aggregate soft OPC UA architecture

S2Fig . OPC UA client and server application architecture

S3Fig. hierarchical structure of workshop information model

S4Fig. Information network architecture of calibration workshop of digital twin smoke alarm

S5Fig. Smoke alarm calibration workshop information model

S6Fig.Mapping of smoke box and information model elements

S7Fig.Smoke box information model

S8Fig.Address space management

S9Fig. Interaction between digital twin model and physical device data

S10Fig. Comparison between digital twin model of cigarette box and real object

S11Fig. System operation verification

S1Table . Mapping of the information model of the smoke chamber

References

1. Tao Fei, Zhang Meng, Cheng Jiang-feng, et al. Digital twin workshop: a new paradigm for future works. Computer Integrated Manufacturing Systems, 2017, 23(1): 1-9
2. Jiuliang, T., Yifan, Y. & Cheng, M. Research on the architecture and application of digital twin system for rocket fight process,Comput Simul,40, 16–19 (2023).
3. Wu, Min; Ying Wenfen.Research on digital twin modeling and monitoring technology for smoke alarm calibration system,SCIENTIFIC REPORTS ,2023.
4. Tao Fei, Liu Wei-ran, Liu Jian-hua, et al. Digital twin and its potential application exploration. Computer Integrated Manufacturing Systems, 2018(1): 1-18
5. WANG Jian, WANG Haochen, LI Xuewei, ZHENG Surui, LI Jiapeng. Digital twin workshop information physical fusion system based on OPC UA, Modern Manufacturing Engineering, 2023(4):43-50
6. Zhong Hua, Zhang Yu, Li Ming, Cha Xianming. Research and Development of OPC UA Bus Bridge Communication System for Intelligent Manufacturing, MACHINE TOOL &HYDRAULICS, 2023(51):109-112
7. Song Tingxin; Li Ke. Data Communication Technology and Applicationins for Intelligent Manufacturing Workshops Based on OPC UA,China Mechanical Engineering,2020(31): 1693-1699

8. Li Feng; Zhang Kun; Yuan Li'na.Research and implementation of information model for textile intelligent dyeing and finishing workshop based on OPC UA,Journal of Textile Research,2020(41):149-154.
9. Wang Min; Cao Pengjun; Song Kaiyu; Zhu Gelong; Zhang Wei; Zhao Qinzhi.Information Interaction Model of Digital Workshop Based on OPC UA for CNC Machine Tools Manufacturing,Journal of Beijing University of Technology,2019(44):1040-1046.
10. Zhou Zheng; Hu Yi; Yang Wei; Su Wenju ; Yu Dong.Research and Implementation of Monitoring Server Architecture of Multi Type CNC Equipment,Journal of Chinese Computer Systems,2021(42):2382-2387.
11. Chen Jiang;Wu Haibo; Zhang Yixiao ; Li Quanfeng.3D real-time monitoring system of workshop operation for digital twinning,Modern Manufacturing Engineering,2023,21-32.
12. IEC TR 62541-1:2016:OPC Unified Architecture-Part 1:Overview and concepts.
13. GB-T 38369-2020 OPC UA- based interconnected network architecture in digital plant.China Standard Publishing House,2021
14. Wang Linkun, ZhaoYanling, Yan Xiaofeng. The research on general solution for information integration of manufacturing equipment in digital workshop, China Instrumentation, 2017(3):21一25
15. Wang Liping;Zhang Zhaokun ;Shao Zhufeng ;Wang Min.Research on the Information Model of Digital Machining Workshop for Machine Tools and Its Applications,Journal of Mechanical Engineering, 2019(55):154-165.
16. Jin Guiyang;Chen Gang;Sun Pingfan.Information model of sweater production workshops based on OPC unified architecture and its application,Journal of Textile Research,2022(43):185-192.
17. Wu Min ;Liu Ping;Ying Wenfeng .Research and Practice of Calibrating Digital Workshop with Smoke Alarm,11th International symposium on project managemen,2023(3):1860-1867.

Figure Click here to access/download;Figure;S1-Fig.pdf

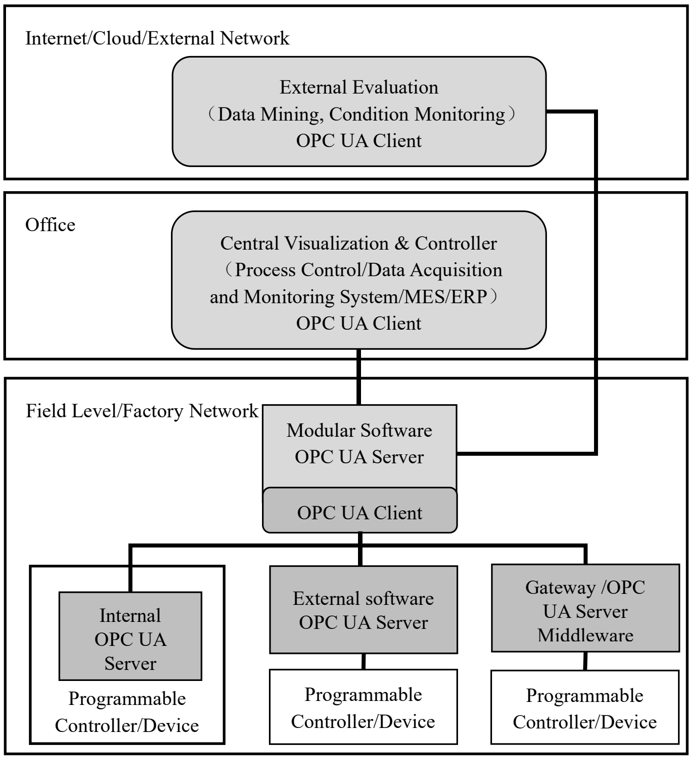

Fig 1 Aggregate soft OPC UA architecture

Figure
Click here to access/download;Figure;S2-Fig.pdf

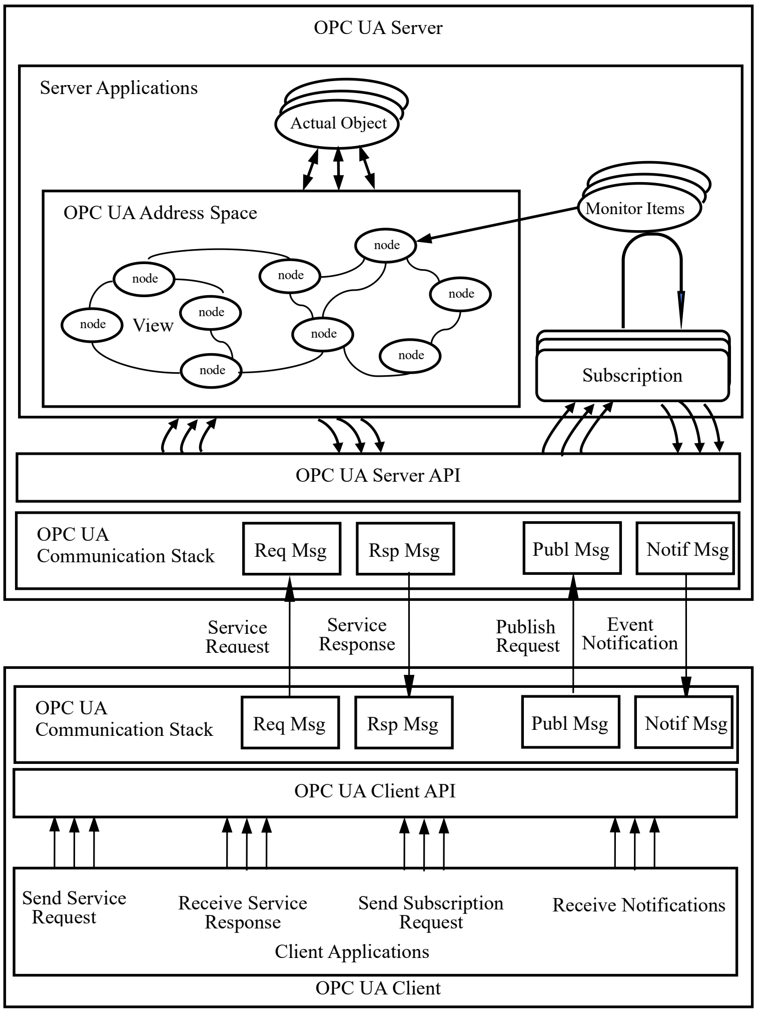

Fig2 OPC UA client and server application architecture

Figure

Click here to access/download;Figure;S3-Fig.pdf

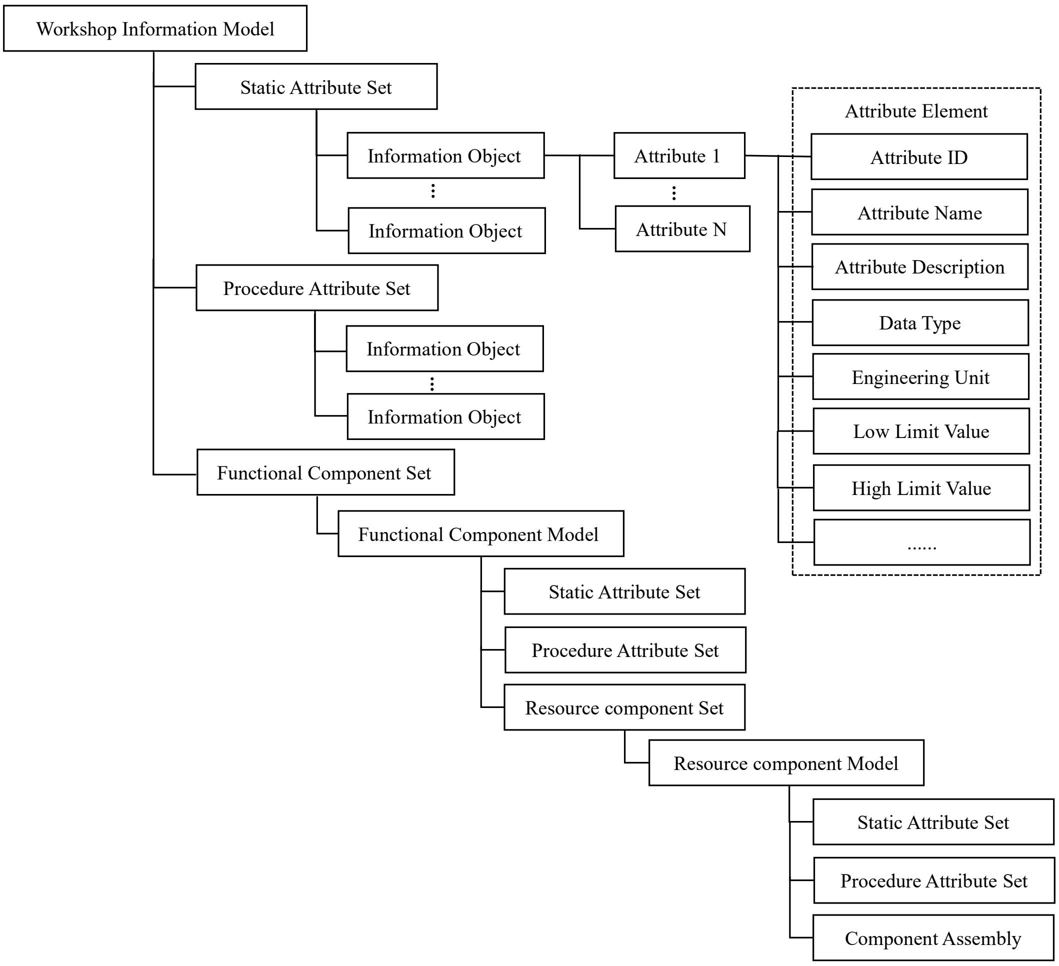

Click here to access/download;Figure;S4-Fig.pdf

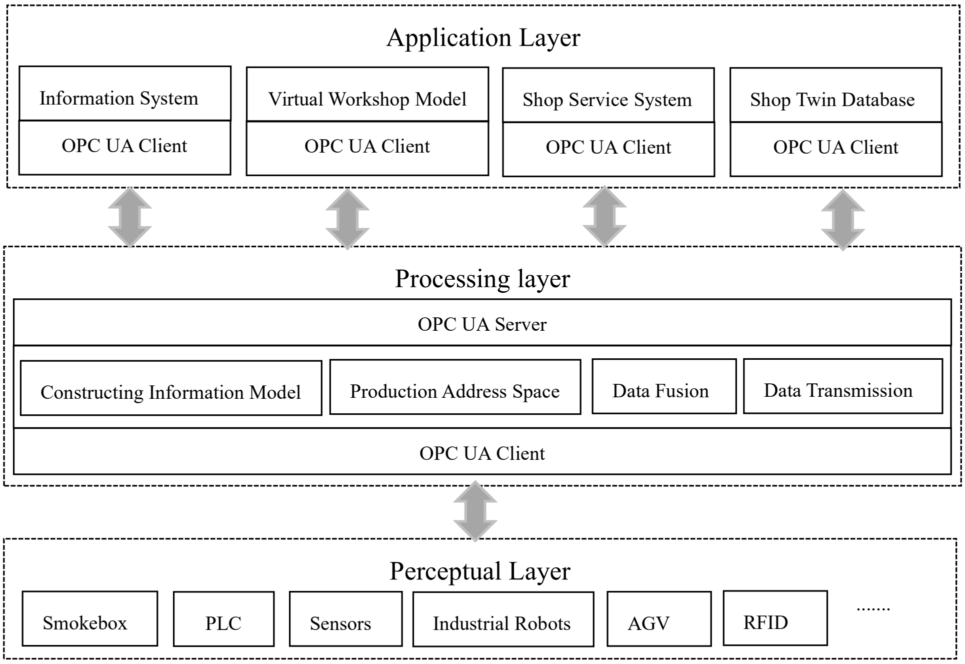

Figure
Click here to access/download;Figure;S5-Fig.pdf

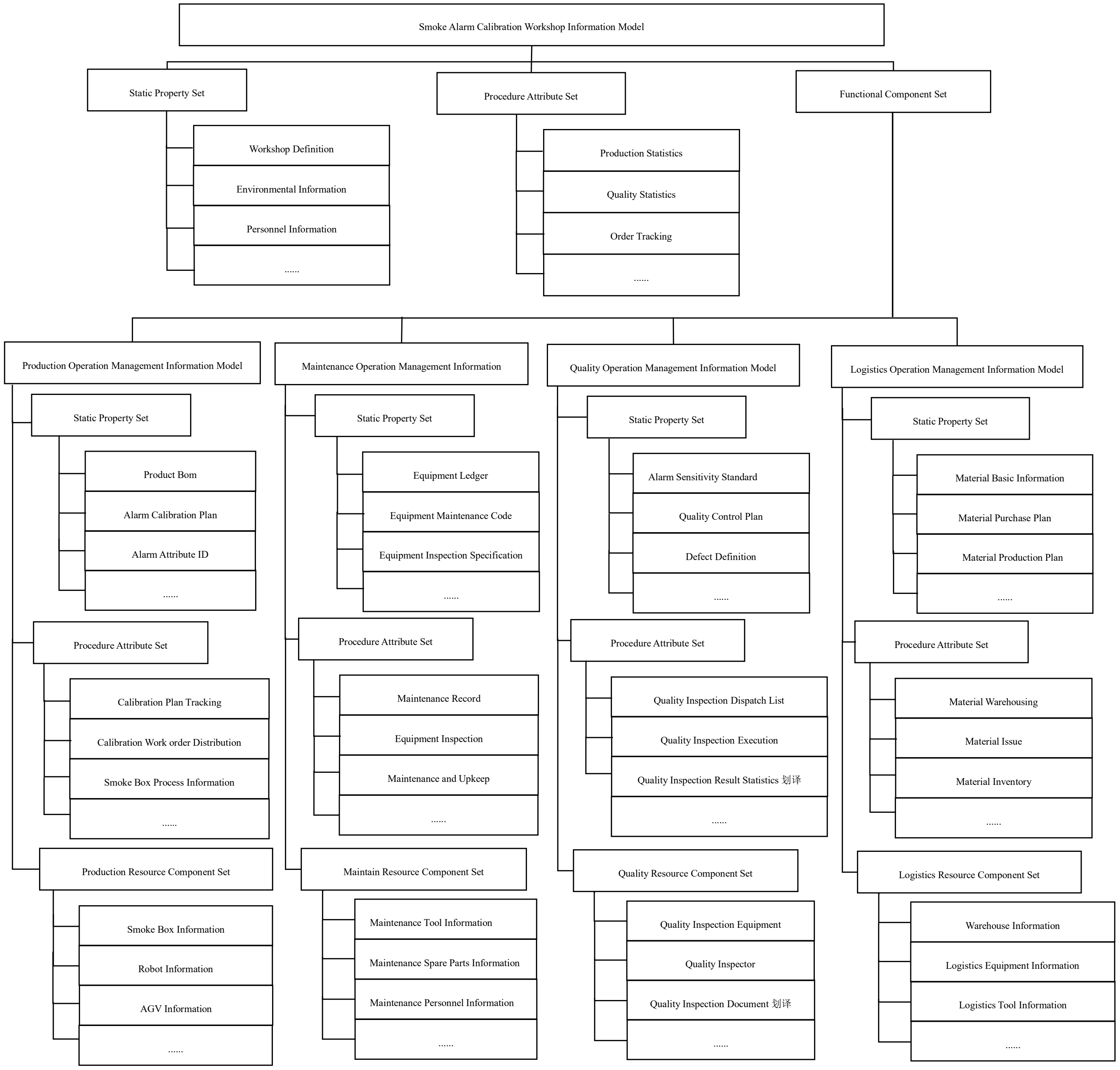

Click here to access/download;Figure;S6-Fig.pdf

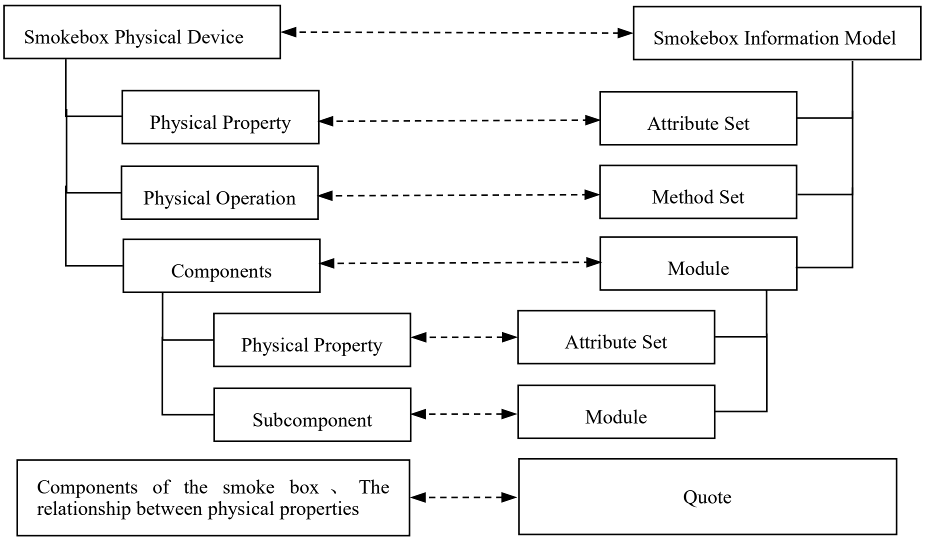

Figure

Click here to access/download;Figure;S7-Fig.pdf

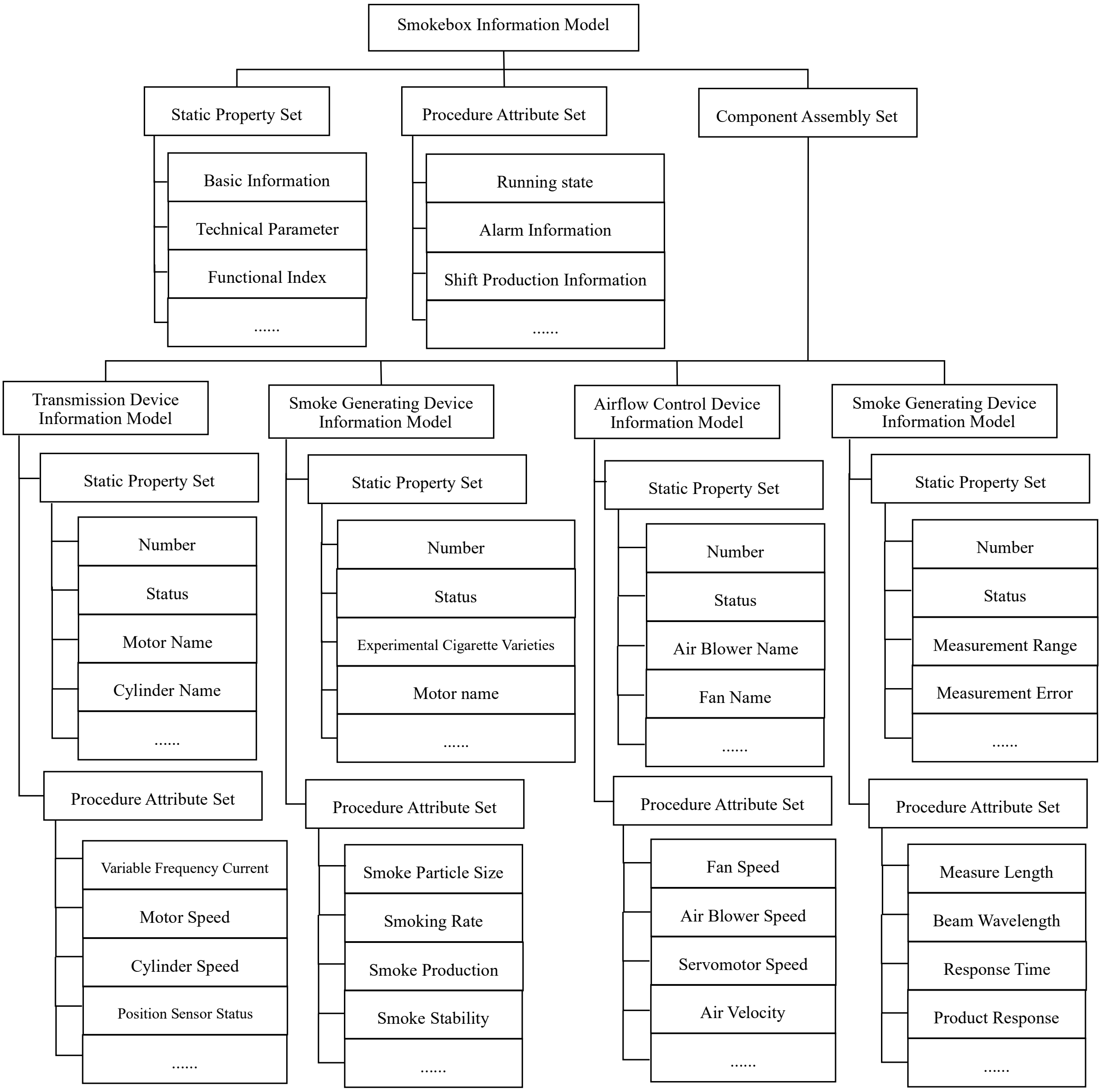

Click here to access/download;Figure;S8-Fig.pdf

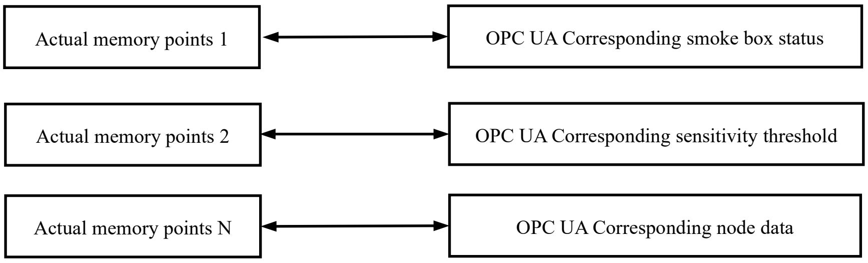

Figure
Click here to access/download;Figure;S9-Fig.pdf

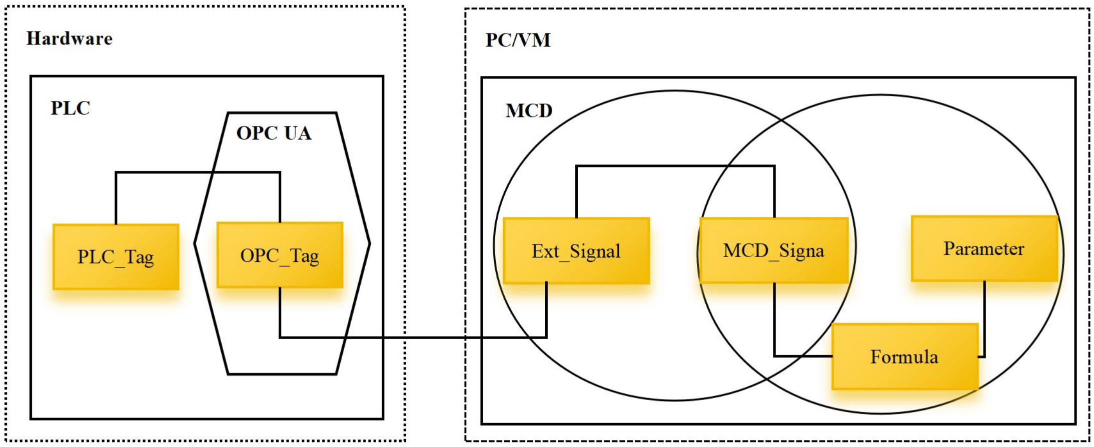

Figure

Click here to access/download;Figure;S10-Fig.pdf

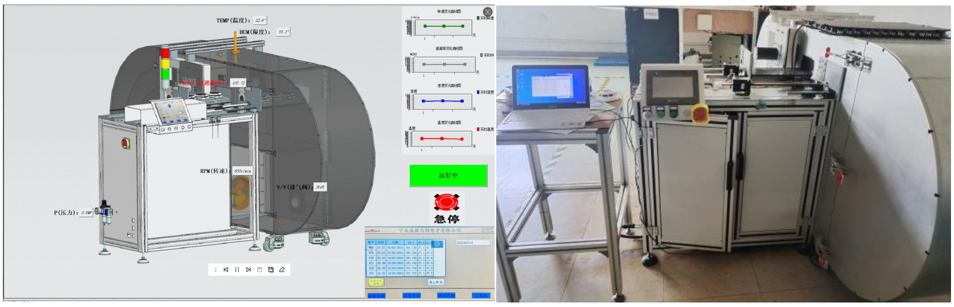

(a) Digital twin model of cigarette box (b)Real cigarette box

Figure Click here to access/download;Figure;S11-Fig.pdf

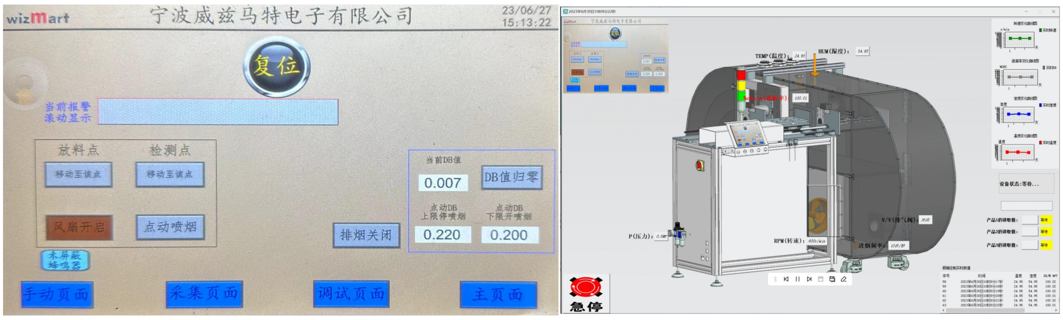

(a) Production management system (b) Twin monitoring interface
