# Supplementary material for: Research and application of OPC UA-based digital twin smoke alarm calibration workshop information model
Source: PLoS One. 2024 Jul 25;19(7):e0307801. doi: 10.1371/journal.pone.0307801 (PMC11271951; doi:10.1371/journal.pone.0307801)
Supplement: Attachment — Submitted filename: Response to the Reviewers comments1.docx [file pone.0307801.s002.docx]

**Dear Editor and Reviewers,**

We thank you very much for giving us another chance to revise and improve our manuscript. We try our best to revise the paper according to the reviewers’ suggestions, and for those comments we have different opinions, we have explained in detail. A list of changes and responses are listed below.

We hope the revised paper has addressed all the reviewers’ concerns and reached the requirements of the journal for publishing.

Thank you very much!

The authors

**Responses to Reviewers’ Comments**

**Reply to reviewer #1**

1. **Concern of the reviewer:**

**When abbreviations first appear in the text, the full term should be provided to facilitate reader understanding.**

**Our response:** We are grateful to the Reviewer for his elaborate work to find out our mistake.We have commented on the first time abbreviations appear in the article

**Revised text:**

**Abstract**

Aiming at the problems of data integration and information sharing in the calibration workshop of smoke alarm, combining OPC Unified Architecture（OPC UA ）and digital twin technology, the OPC UA based digital twin smoke alarm calibration workshop information network framework is proposed. Using OPC UA information modeling technology, a digital workshop information model for smoke alarm calibration was constructed, and the static attribute set, process attribute set, and functional component set of the workshop were defined in detail. Taking the smoke box as an example, the information model construction was instantiated, and data collection and transmission were carried out with the digital twin model of the smoke box, achieving the implementation mapping between the twin model and physical entities. The results show that based on the proposed OPC UA information model, the interconnection and intercommunication of data at all levels of the digital twin smoke alarm calibration workshop can be achieved, and the workshop can be visualized, digitized, and flexible.

**Introduction**

With the successive propositions of strategies such as Industry 4.0 and Made in China 2025, intelligent manufacturing has become the core of development for countries at the advent of the fourth industrial revolution. As the best means of integrating interactive physical world and virtual world, digital twin is a major driving force to promote the further transformation of manufacturing workshop towards intelligence, personalization and service[1]. With the increasing demand for smoke alarms, their quantity and variety are also increasing. The calibration workshop of smoke alarms will generate massive data during production and operation. How to use these data to establish a multi-domain integrated data model and achieve an efficient application model of real-time interaction between data model and physical entity is a problem to be solved in the production process of smoke alarms[2]. Digital twin technology provides a possibility to solve this problem[3].

The core of digital twinning is modeling and data interaction[4]. Designing a reasonable information-physical fusion system for digital twinning workshop is the foundation for building virtual-real interaction in digital workshop[5], and it is also the key to solve the problem of information island in digital workshop.OPC Unified Architecture (OPC UA) protocol is one of the current common data transmission standards in the industrial field, which realizes the semantic unification of data between various layers in intelligent manufacturing workshop[6]. Regarding the application of OPC UA technology, Song,et al. [7] implemented data integration and unified management of intelligent workshop equipment information based on OPC UA technology. Li,et al. [8] proposed a textile intelligent dyeing and finishing workshop information model scheme based on OPC UA technology, which realized data communication between equipment end and

2. **Concern of the reviewer:**

**The review of the application of OPC UA in the field of smoke alarms is not comprehensive enough and should be supplemented with the current status of relevant technologies in this field.**

**Our response:** Thanks very much to the Reviewer for his meticulous work to find out our weakness , We have added a review of OPC UA applications in the field of smoke alarms.

**Revised text:** The application research of OPC UA in smoke alarm related fields was conducted by searching keywords "smoke alarm," "smoke detector," or "smoke sensor" in CNKI, Wanfang Data, Web of Science, Scopus, Baidu Library and other databases. The retrieved articles mainly focused on smoke alarm design, smoke alarm detection system design, and smoke alarm monitoring technology. However, there is a lack of scholarly research on the production aspect of smoke alarms. A search using the keywords "smoke alarm and OPC UA" or "smoke detector and OPC UA" or "smoke sensor and OPC UA" yielded no results. It is evident that there is a gap in data information related to the production workshop of smoke alarms. Therefore, it is crucial to optimize the production process and strengthen workshop management research for smoke alarm manufacturing enterprises.

3. **Concern of the reviewer:**

**Conceptual introductions in the text should be accompanied by references.**

**Our response:** We are grateful to the Reviewer for his elaborate work to find out our mistake.We have attached references to the concepts in the text.

**Revised text:**

**1 OPC UA information modeling technology**

The OPC Unified Architecture provides a standardized, synchronous or asynchronous, and distributed communication mechanism[12]. Under this mechanism, it allows access to different types of data horizontally or vertically. OPC UA components can be combined in different forms, on different platforms, and by different vendors[13]. Their scale can range from embedded OPC UA components in devices or controllers, to machines or complete sets of equipments using network management to provide OPC UA functionality, all the way to OPC UA server clusters[14]. OPC UA is generally based on a distributed client-server model[15].

**1.1 OPC UA Server**

According to the functional requirements of the digital twin smoke alarm calibration workshop, an aggregated soft OPC UA server architecture is adopted, as shown in Fig1. The aggregated OPC UA server unifies the OPC UA servers for each asset, incorporates the information models of all servers in the lower layer, and integrates them at the system aggregation level[16]. This architecture type can be connected to enterprise MES or ERP, or used to separate manufacturing systems from later data analysis or earlier planning[17]. The OPC UA client can be either a SCADA, MES, ERP, or central control system maintained by the IT department, a visualization terminal running near the machine, or even an external application[18].

12.Gu Xiaohong,Hu Yang,Chen Xu,et al.Design and development of water system data acquisition system based on OPC UA.Industrial Control Computer.2022;35(6):114-118.

13.Zhong Hua,ZhangYu,Li Ming,et al.Research and development of OPC UA bus bridge comunication system for intelligent manufacturing.Machine Tool & Hydraulics,2023;51(9):109-112.

14.[Axel Busboom](https://scholar-cnki-net-s.webvpn.nbpt.edu.cn/home/search?sw=6&sw-input=Axel Busboom" \t "https://kns-cnki-net-s.webvpn.nbpt.edu.cn/kcms2/article/_blank).Automated generation of OPC UA information models -A review and outlook.[Journal of Industrial Information Integration.](https://scholar-cnki-net-s.webvpn.nbpt.edu.cn/journal/index/SJES2452414X3545)2024(39):100602-100613.

15.[Andreas Lober](https://scholar-cnki-net-s.webvpn.nbpt.edu.cn/home/search?sw=6&sw-input=Andreas Lober" \t "https://kns-cnki-net-s.webvpn.nbpt.edu.cn/kcms2/article/_blank),[Joel Lehmann](https://scholar-cnki-net-s.webvpn.nbpt.edu.cn/home/search?sw=6&sw-input=Joel Lehmann" \t "https://kns-cnki-net-s.webvpn.nbpt.edu.cn/kcms2/article/_blank),[Jakob Weber](https://scholar-cnki-net-s.webvpn.nbpt.edu.cn/home/search?sw=6&sw-input=Jakob Weber" \t "https://kns-cnki-net-s.webvpn.nbpt.edu.cn/kcms2/article/_blank),et al.Agentenbasiertes Redesign und Neuinterpretation von OPC UA Designstrategien zur Flexiblen Fähigkeitsbasierten Produktion.[at - Automatisierungstechnik.](https://scholar-cnki-net-s.webvpn.nbpt.edu.cn/journal/index/SJDG017823120071)2024;72(3):233-259.

16.[Martins André](https://scholar-cnki-net-s.webvpn.nbpt.edu.cn/home/search?sw=6&sw-input=Martins André" \t "https://kns-cnki-net-s.webvpn.nbpt.edu.cn/kcms2/article/_blank),[Lucas João](https://scholar-cnki-net-s.webvpn.nbpt.edu.cn/home/search?sw=6&sw-input=Lucas João" \t "https://kns-cnki-net-s.webvpn.nbpt.edu.cn/kcms2/article/_blank),[Costelha Hugo](https://scholar-cnki-net-s.webvpn.nbpt.edu.cn/home/search?sw=6&sw-input=Costelha Hugo" \t "https://kns-cnki-net-s.webvpn.nbpt.edu.cn/kcms2/article/_blank),[Neves Carlos](https://scholar-cnki-net-s.webvpn.nbpt.edu.cn/home/search?sw=6&sw-input=Neves Carlos" \t "https://kns-cnki-net-s.webvpn.nbpt.edu.cn/kcms2/article/_blank).Developing an OPC UA srver for CNC machines.[Procedia Computer Science](https://scholar-cnki-net-s.webvpn.nbpt.edu.cn/journal/index/SJES187705092816).2021(180):561-570.

17.[Bauer Heiner](https://scholar-cnki-net-s.webvpn.nbpt.edu.cn/home/search?sw=6&sw-input=Bauer Heiner" \t "https://kns-cnki-net-s.webvpn.nbpt.edu.cn/kcms2/article/_blank),[Hoeppner Sebastian](https://scholar-cnki-net-s.webvpn.nbpt.edu.cn/home/search?sw=6&sw-input=Hoeppner Sebastian" \t "https://kns-cnki-net-s.webvpn.nbpt.edu.cn/kcms2/article/_blank),[Iatrou Chris](https://scholar-cnki-net-s.webvpn.nbpt.edu.cn/home/search?sw=6&sw-input=Iatrou Chris" \t "https://kns-cnki-net-s.webvpn.nbpt.edu.cn/kcms2/article/_blank),et al.Hardware implementation of an OPC UA server for industrial field devices.[IEEE Transactions on very large scale integration (VLSI) systems](https://scholar-cnki-net-s.webvpn.nbpt.edu.cn/journal/index/SBQK106382101451).2021;29(11):1998-2002.

18.[Iatrou C.P.](https://scholar-cnki-net-s.webvpn.nbpt.edu.cn/home/search?sw=6&sw-input=Iatrou C.P." \t "https://kns-cnki-net-s.webvpn.nbpt.edu.cn/kcms2/article/_blank),[Ketzel L.](https://scholar-cnki-net-s.webvpn.nbpt.edu.cn/home/search?sw=6&sw-input=Ketzel L." \t "https://kns-cnki-net-s.webvpn.nbpt.edu.cn/kcms2/article/_blank),[Urbas L.](https://scholar-cnki-net-s.webvpn.nbpt.edu.cn/home/search?sw=6&sw-input=Urbas L." \t "https://kns-cnki-net-s.webvpn.nbpt.edu.cn/kcms2/article/_blank),et al.Classification and methodical design of OPC UA aggregating servers for smart architecture.[VDI Berichte.](https://scholar-cnki-net-s.webvpn.nbpt.edu.cn/journal/index/SJPD008355603755)2020;781-796.

4. **Concern of the reviewer:**

**Section 3.1 claims that a "digital twin smoke alarm calibration workshop information model" was established; the basis for the architecture's establishment should be described in detail.**

**Our response:** We are grateful to the Reviewer for his elaborate work to find out our weakness.

We have added the foundation for building the "Digital Dual Smoke Alarm Calibration shop Information Model" architecture.According to the journal manuscript template requirements, the chapters are reordered, 3.1 is changed to 2.1.

**Revised text:**

**2.1 Smoke alarm calibration workshop architecture**

The smoke alarm calibration workshop is structured with a management layer, an execution layer, and a base layer, as illustrated in Figure 3. The management layer primarily comprises enterprise resource planning systems and product life cycle management systems to address workshop management issues. The execution layer encompasses production process management, production process quality management, production scheduling management, equipment management, order management, logistics management, and manufacturing operation management to oversee various business activities and related assets in the production process. The base layer consists of AGV cars, smoke boxes, industrial robots, intelligent printers, and intelligent workstations.

According to GB/T7393-2019 "General Technology of Digital Workshop", the essential functionalities of a digital workshop should encompass workshop planning and scheduling, production logistics management, process execution and management, workshop equipment management, and production process quality control. In conjunction with the requirements of smoke alarm calibration process, the data flow between functional modules of smoke alarm calibration workshop is illustrated in Figure 4.

**Fig4.Digital workshop data flow diagram[21]**

According to the organizational structure, functional modules and data flow requirements of the smoke alarm calibration workshop, the physical equipment and calibration process data were digitized and modeled. They were then organized according to information modeling rules to form a digital smoke alarm workshop information model. The hierarchical architecture of the workshop information model is illustrated in Figure 5. The workshop information model corresponds to the entire workshop, while the functional component set corresponds to each functional module of the workshop, and the resource component set represents all types of resources involved in each module. The attribute set represents a collection of attributes with a fixed structure that describes the correlation between devices, components, attribute sets, and attributes [22].

**Fig5. Workshop information model hierarchy**

5. **Concern of the reviewer:**

**The author states, "Through testing and verification of the digital twin system, the calibration process of the smoke box is visually monitored." The effects of the digital twin system should be specifically demonstrated.**

**Our response:** We are grateful to the Reviewer for his elaborate work to find out our weakness.

We add that the effects of digital twin systems in section 3.4

**Revised text:**

**3.4 Application of information model**

This article uses Siemens NX platform to construct a digital twin model for the smoke box, builds a virtual-reality communication platform using OPC UA communication protocol, and finally uses real-time mapping technology to realize 3D model visualization and virtual production visualization. Siemens NX is equipped with an OPC UA client, which can realize data exchange between the digital twin model's data, signals, and variables and external servers. The entire environment consists of three main components: PLC CPU, OPC server and digital twin model (MCD). The CPU is used to process the logic of operation, obtain the machine state through the OPC server, and send operating instructions to the device through the OPC server; MCD is used to display the virtual model and perform simulation, obtain CPU instructions from OPC, and feedback the current device state through the OPC server. The data interaction between the digital twin model and physical equipment is shown in Fig11[3], and Fig12 is a comparison diagram of the digital twin model of the smoke box and the physical object.

**Fig11. Data interaction between digital twin model and physical equipment**

(a) Digital twin model of cigarette box	(b)Real cigarette box

**Fig12.Comparison between the digital twin model of the smoke chamber and the physical object**

In the application experiment, a specific model of smoke alarm was chosen to follow its complete lifecycle workflow at the calibration site, spanning from order issuance, production scheduling, product dispatch, calibration procedure, calibration execution, quality inspection, to finished product warehousing. By connecting to the backend data server via Web Service, the updates and modifications in the respective data tables were monitored, thereby verifying the synchronized updating of the information organization within the information model. The detailed verification process is depicted in Figure 13.

**Fig13.Flow chart of field application test**

The production process information management system of the workshop is illustrated in Figure 14. During the calibration process, the digital twin system displays the real-time operational data and the data change curves for various parameters of the smoke detector. The calibration data for the smoke detector is saved in the database.

**Fig14.Production process information management system**

The application of digital twin smoke alarm workshop has broken the traditional manual management mode of low efficiency, error-prone, manual record and paper transmission, and solved the drawbacks such as no display of field detection equipment information, no statistics of detection information, no traceability of quality problems, and delay of manual scheduling production information transmission. It realizes the visualization of field equipment information, information statistics, on-time production scheduling, traceability of quality problems, paperless process management, networked data transmission, and intelligent field management. After the digital twin workshop was put into operation, the production efficiency was increased by 200 percent, and the pass rate was increased from 98 percent to 99.6 percent. The annual production capacity of a single unit was increased from 80W to 160W, and the overall production capacity of 10 units was increased from 800W to 1600W. The repair rate of defective products was reduced by 1.6%, indirectly recovering the economic loss of 51.2W.

6. **Concern of the reviewer:**

**Chinese labels in the images should be provided with English annotations.**

**Our response:**Thank you for the feedback provided by Reviewer .We have changed the content of the image to English.

**Reply to reviewer #2**

**1.Concern of the reviewer:**

**It is best to label and have a title below the figures appearing in the manuscript for easy reference by readers.**

**Our response:**We are grateful to the Reviewer for his elaborate work to find out our weakness.We put labels and titles below the figures .

**2.Concern of the reviewer:**

**The entries in the references should have a unified format.**

**Our response:**We are grateful to the Reviewer for his elaborate work to find out our mistake.We have re-edited the references according to the manuscript template.

**Revised text:**

1. Tao Fei, Zhang Meng, Cheng Jiangfeng, et al. Digital twin workshop: a new paradigm for future works. Computer Integrated Manufacturing Systems. 2017;23(1): 1-9.
2. Tao Jiuliang, Yu Yifan, Ma Cheng.Research on architecture and application of digital twin system for fight of launch rocket.Computer Simulation.2023;40(3), 16-19.
3. Wu Min, Ying Wenfen.Research on digital twin modeling and monitoring technology for smoke alarm calibration system.Scientific Reports.2023;13(1):19239.
4. Tao Fei, Liu Weiran, Liu Jian-hua, et al. Digital twin and its potential application exploration. Computer Integrated Manufacturing Systems.2018(1): 1-18.
5. Wang Jian, Wang Haochen, LI Xuewei, et al. Digital twin workshop information physical fusion system based on OPC UA.Modern Manufacturing Engineering.2023(4):43-50.
6. Zhong Hua,Zhang Yu,Li Ming,et al.Research and Development of OPC UA Bus Bridge Communication System for Intelligent Manufacturing. Machine Tool＆Hydraulics. 2023(51):109-112.
7. Song Tingxin, Li Ke. Data communication technology and applicationins for intelligent manufacturing workshops based on OPC UA.China Mechanical Engineering.2020(31): 1693-1699.
8. Li Feng, Zhang Kun,Yuan Lina.Research and implementation of information model for textile intelligent dyeing and finishing workshop based on OPC UA.Journal of Textile Research.2020(41):149-154.
9. Wang Min,Cao Pengjun,Song Kaiyu,et al.Information Interaction Model of Digital Workshop Based on OPC UA for CNC Machine Tools Manufacturing.Journal of Beijing University of Technology.2019(44):1040-1046.
10. Zhou Zheng,Hu Yi,Yang Wei,et al.Research and implementation of monitoring server architecture of multi type CNC equipment,Journal of Chinese Computer Systems.2021(42):2382-2387.
11. Chen Jiang,Wu Haibo,Zhang Yixiao, et al.3D real-time monitoring system of workshop operation for digital twinning.Modern Manufacturing Engineering.2023(4):21-32.
12. Gu Xiaohong,Hu Yang,Chen Xu,et al.Design and development of water system data acquisition system based on OPC UA.Industrial Control Computer.2022;35(6):114-118.
13. Zhong Hua,ZhangYu,Li Ming,et al.Research and development of OPC UA bus bridge comunication system for intelligent manufacturing.Machine Tool & Hydraulics,2023;51(9):109-112.
14. [Axel Busboom](https://scholar-cnki-net-s.webvpn.nbpt.edu.cn/home/search?sw=6&sw-input=Axel Busboom" \t "https://kns-cnki-net-s.webvpn.nbpt.edu.cn/kcms2/article/_blank).Automated generation of OPC UA information models -A review and outlook.[Journal of Industrial Information Integration.](https://scholar-cnki-net-s.webvpn.nbpt.edu.cn/journal/index/SJES2452414X3545)2024(39):100602-100613.

### 15. [Andreas Lober](https://scholar-cnki-net-s.webvpn.nbpt.edu.cn/home/search?sw=6&sw-input=Andreas Lober" \t "https://kns-cnki-net-s.webvpn.nbpt.edu.cn/kcms2/article/_blank),[Joel Lehmann](https://scholar-cnki-net-s.webvpn.nbpt.edu.cn/home/search?sw=6&sw-input=Joel Lehmann" \t "https://kns-cnki-net-s.webvpn.nbpt.edu.cn/kcms2/article/_blank),[Jakob Weber](https://scholar-cnki-net-s.webvpn.nbpt.edu.cn/home/search?sw=6&sw-input=Jakob Weber" \t "https://kns-cnki-net-s.webvpn.nbpt.edu.cn/kcms2/article/_blank),et al.Agentenbasiertes Redesign und Neuinterpretation von OPC UA Designstrategien zur Flexiblen Fähigkeitsbasierten Produktion.[at - Automatisierungstechnik.](https://scholar-cnki-net-s.webvpn.nbpt.edu.cn/journal/index/SJDG017823120071)2024;72(3):233-259.

### 16. [Martins André](https://scholar-cnki-net-s.webvpn.nbpt.edu.cn/home/search?sw=6&sw-input=Martins André" \t "https://kns-cnki-net-s.webvpn.nbpt.edu.cn/kcms2/article/_blank),[Lucas João](https://scholar-cnki-net-s.webvpn.nbpt.edu.cn/home/search?sw=6&sw-input=Lucas João" \t "https://kns-cnki-net-s.webvpn.nbpt.edu.cn/kcms2/article/_blank),[Costelha Hugo](https://scholar-cnki-net-s.webvpn.nbpt.edu.cn/home/search?sw=6&sw-input=Costelha Hugo" \t "https://kns-cnki-net-s.webvpn.nbpt.edu.cn/kcms2/article/_blank),[Neves Carlos](https://scholar-cnki-net-s.webvpn.nbpt.edu.cn/home/search?sw=6&sw-input=Neves Carlos" \t "https://kns-cnki-net-s.webvpn.nbpt.edu.cn/kcms2/article/_blank).Developing an OPC UA srver for CNC machines.[Procedia Computer Science](https://scholar-cnki-net-s.webvpn.nbpt.edu.cn/journal/index/SJES187705092816).2021(180):561-570.

### 17. [Bauer Heiner](https://scholar-cnki-net-s.webvpn.nbpt.edu.cn/home/search?sw=6&sw-input=Bauer Heiner" \t "https://kns-cnki-net-s.webvpn.nbpt.edu.cn/kcms2/article/_blank),[Hoeppner Sebastian](https://scholar-cnki-net-s.webvpn.nbpt.edu.cn/home/search?sw=6&sw-input=Hoeppner Sebastian" \t "https://kns-cnki-net-s.webvpn.nbpt.edu.cn/kcms2/article/_blank),[Iatrou Chris](https://scholar-cnki-net-s.webvpn.nbpt.edu.cn/home/search?sw=6&sw-input=Iatrou Chris" \t "https://kns-cnki-net-s.webvpn.nbpt.edu.cn/kcms2/article/_blank),et al.Hardware implementation of an OPC UA server for industrial field devices.[IEEE Transactions on very large scale integration (VLSI) systems](https://scholar-cnki-net-s.webvpn.nbpt.edu.cn/journal/index/SBQK106382101451).2021;29(11):1998-2002.

### 18. [Iatrou C.P.](https://scholar-cnki-net-s.webvpn.nbpt.edu.cn/home/search?sw=6&sw-input=Iatrou C.P." \t "https://kns-cnki-net-s.webvpn.nbpt.edu.cn/kcms2/article/_blank),[Ketzel L.](https://scholar-cnki-net-s.webvpn.nbpt.edu.cn/home/search?sw=6&sw-input=Ketzel L." \t "https://kns-cnki-net-s.webvpn.nbpt.edu.cn/kcms2/article/_blank),[Urbas L.](https://scholar-cnki-net-s.webvpn.nbpt.edu.cn/home/search?sw=6&sw-input=Urbas L." \t "https://kns-cnki-net-s.webvpn.nbpt.edu.cn/kcms2/article/_blank),et al.Classification and methodical design of OPC UA aggregating servers for smart architecture.[VDI Berichte.](https://scholar-cnki-net-s.webvpn.nbpt.edu.cn/journal/index/SJPD008355603755)2020;781-796.

19. IEC TR 62541-1:2016:OPC Unified Architecture-Part 1:Overview and concepts.
20. GB-T 38369-2020 OPC UA- based interconnected network architecture in digital plant.China Standard Publishing House.2021.
21. Wu Min ,Liu Ping,Ying Wenfeng .Research and practice of calibrating digital workshop with smoke alarm.11th International symposium on project managemen.2023(3):1860-1867.
22. Wang Linkun,ZhaoYanling,Yan Xiaofeng. The research on general solution for information integration of manufacturing equipment in digital workshop.China Instrumentation. 2017(3):21-25
23. Wang Liping,Zhang Zhaokun,Shao Zhufeng,Wang Min.Research on the information model of digital machining workshop for machine tools and its applications.Journal of Mechanical Engineering.2019(55):154-165.
24. Jin Guiyang,Chen Gang,Sun Pingfan.Information model of sweater production workshops based on OPC unified architecture and its application.Journal of Textile Research.2022(43):185-192.

**3.Concern of the reviewer:**

**Some minor errors, including spelling, grammar, punctuation, etc., have been marked in the manuscript as attached.**

**Our response:** We are grateful to the Reviewer for his elaborate work to find out our mistake.We corrected the marks in the manuscript.
